# Role of TMPRSS4 Modulation in Breast Cancer Cell Proliferation

**DOI:** 10.31557/APJCP.2019.20.6.1849

**Published:** 2019

**Authors:** Ganiou Assani, Akadiri Yessoufou, Yudi Xiong, Julien Segbo, Xiaoyan Yu, Fuxiang Zhou, Yunfeng Zhou

**Affiliations:** 1 *Hubei Cancer Clinical Study Center, Hubei Key Laboratory of Tumor Biological Behaviors, *; 2 *Department of Radiation and Medical Oncology, Zhongnan Hospital, Wuhan University, Wuhan, Hubei, China, *; 3 *University of Abomey Calavi, BP 526, Cotonou, Benin. *

**Keywords:** TMPRSS4, breast cancer, cell cycle, cell apoptosis, Telomere

## Abstract

**Background:**

TMPRSS4 is a novel Type II transmembrane serine protease found at the surface of the cells and is involved in the development and cancer progression. However, TMPRSS4 functions in breast cancer remain poor understand. The present study investigated the function of TMPRSS4 in the breast cancer cells and the potential mechanistic action underling.

**Materials and Methods:**

The lentiviral vectors causing TMPRSS4 down-regulation and over-expression were established and transfected in MDA-MB-468 and MCF-7 cells, respectively. By using the CCK-8 assay, cell proliferation was analyzed. Moreover, western blot was used to detect the expression of certain proteins related to cell apoptosis (Bax and Bcl2) signaling pathway and telomere maintenance (POT1, TPP1, and UBE2D3). Cell cycle and cell apoptosis were also analyzed by using the Flow cytometry analysis. TMPRSS4 expression was detected at the mRNA level and protein level by performing qPCR and western blot technique, respectively.

**Results:**

TMPRSS4 expression is inhibited in stable transfected MDA-MB-468-shTMPRSS4 cells compared to the control MDA-MB-468-NC and its expression is up-regulated in stable transfected MCF-7-TMPTSS4 compared to its control MCF-7-NC. Moreover, TMPRSS4 silencing in breast cancer reduces cells proliferation by promoting cell cycle arrest in G2/M phase, cell apoptosis, and telomere maintenance impairment while the TMPRSS4 overexpression increases cells proliferation through cell apoptosis reduction and telomere maintenance reinforcement associated with insignificant change in cell cycle progression.

**Conclusion:**

TMPRSS4 plays important roles in cancer progression and may be considered as a good therapeutic target for cancer gene therapy especially breast cancer.

## Introduction

Breast cancer is the most common neoplasm diagnosis among women worldwide and is the leading cause of female cancer death (Jemal et al., 2011). In 2015, 2.4 million incident cases and 523,000 death cases were estimated worldwide and the increase in the number is predicted in the future (Bellanger et al., 2018; Linos et al., 2008). The Cancer gene therapy (CGT) which is the modulation of genes implicated in cancer progression, is one of the new cancer treatment strategies developed and used nowadays (Wirth et al., 2014; Lui et al., 2018). Moreover, the Type II Transmembrane serine proteases (TTPs) are a subfamily of serine proteases with a common proteolytic domain, a transmembrane domain, a short cytoplasmic domain and variable length stem region (Szabo et al., 2003). TTPs are implicated in the regulation of cellular signaling, tumor initiation, and progression via itself deregulation (Netzel Arnett et al., 2003; Hooper et al., 2001; Choi et al.,2009 ). TMPRSS4 is a member of TTPs and is often overexpressed in many types of cancer tissues such as pancreatic, thyroid, colon, lung, gastric, breast and other cancer tissues, with a poor clinical prognostic (Kim et al.,2014; Chikaishi et al., 2016; Wu et al., 2014; Liang et al., 2013; Cheng et al., 2013). TMPRSS4 is implicated in cell mobility, invasion, proliferation and tumor metastasis (Kim et al., 2010; Larzabal et al., 2014). It has been reported that TMPRSS4 down-regulation suppressed cell proliferation rate (Fan et al., 2018; Huang et al., 2014; Lee et al., 2016; Jung et al., 2008), induced cell apoptosis (Fan et al., 2018; Huang et al., 2014), promotes cell cycle arrest (Lee et al., 2016; Jung et al., 2008), and cell migration and invasion (Lee et al., 2016; Jung et al., 2008; Min et al., 2014; Min et al., 2014). Moreover, telomeres are DNA proteins complexes found at the ends of eukaryotic chromosomes for the protection of these chromosomes ends from its recognition as chromosome breaks. Loss of telomere protection ( telomere maintenance) activates DNA damage like signaling response which can halt cell proliferation or promote cell death via induction of tumor suppressors P53 and p16 (De Lange et al., 2010; O’Sullivan et al., 2010 ). However, the effect of TMPRSS4 on cell proliferation in relationship with telomere integrity remains unclear, especially in breast cancer. We undertook this study to confirm in breast cancer, the predicted roles of cell cycle and cell apoptosis in TMPRSS4 modulation mediated cell proliferation modulation found before in other types of cancer cell lines and to investigate the effect of TMPRSS4 modulation on cell proliferation in relationship with telomere integrity. From this study, we showed that cell apoptosis and telomeres are implicated in TMPRSS4 expression modulation induced modulation of cells proliferation while cell cycle is only implicated in TMPRSS4 expression silencing reduced cell proliferation in breast cancer. 

## Materials and Methods

*Cell line, cell culture, and reagents*

MDA-MB-468 cells and MCF-7 cells were obtained from the key laboratory of Tumor Biological Behavior of Hubei Province and incubated under 5% CO_2_ at 37^o^C in DMEM (Hyclone, USA) containing 10% fetal bovine serum (WiSant, Canada), 100 U/mL penicillin and 100 ug/mL streptomycin.

*Vector, transfection, and generation of stable cell lines*

Human breast cancer cells MDA-MB-468 were stably transfected with TMPRSS4 down-regulating vectors PLKO.1-sh-TMPRSS4 versus PLKO.1-sh-NC. The full-length cDNA encoding TMPRSS4 was cloned into the lentiviral vector pHAGE-CMV-MCS-PGK-puro N-HA+C-Flag followed by its transfection in MCF-7 cells and the similar plasmid was used to establish a control clone (Bioeagle/Wuhan/China). Puromycin-resistant clones were selected at 5 ug/mL puromycin after two to three weeks post-transfection. The resultant stably transfected cell lines were respectively called MDA-MB-468-sh-TMPRS4 versus MDA-MB-468-sh-NC and MCF-7-TMPRSS4 versus MCF-7-NC. Quantitative PCR and western blot were used to analyze, in resultant stably transfected cell lines, the expression of TMPRSS4 at mRNA and protein level, respectively.

*Quantitative PCR (q-PCR) and RNA isolation*

Total RNA was isolated from the resultant stably transfected MDA-MB-468 and MCF-7 cell lines by using TRIzol reagent according to the manufacturer’s protocol, followed by measurement of the RNA concentration. cDNA was generated from extracted total RNA by using the HiScript qRT SuperMix (Thermo Scientific, Waltham, MA, USA), for 15 min at 50^o^C. The resultants cDNA were amplified using the RevertAid™ First-Strand cDNA Synthesis kit (Fermentas International, Inc., Burlington, ON, Canada). For the quantitative analysis, the glyceraldehyde-3-phosphate dehydrogenase (GAPDH) was used as an internal control. The nucleotide sequence or specific primer for mRNA were designed as follows: TMPRSS4 Sense, 5′-CCGATGTGTTCAACTGGAAG-3′ and anti-sense, 5′-GAGAAAGTGAGTGG GAACTG-3′; GAPDH sense, 5’-GCACCGTCAAGGCTGAGAAC-3′ and anti-sense, 5’-TGGTGAAGACGCCAGTGGA-3’. After denaturation for 5 min at 950C, the reaction was conducted with 40 cycles of 45 sec at 94^o^C, for 45 sec at 55^o^C, 60 sec at 72^o^C and a final extension for 7 min at 72^o^C. The PCR products were identified using electrophoresis on 1.5% agarose gels containing 0.5% ethidium bromide (ED). The thermal amplification was performed on Mx3000P qPCR System (Stratagene, La Jolla, CA, USA) and the Mx3000P analysis program was used to analyze the results. All experiments were carried out in triplicate.

*Western blot analysis*

The resultant stably transfected MDA-MB-468 and MCF-7 cells were treated with RIPA lysis buffer (#P0013B, Beyotime biotechnology,China) with an addition of 1xPMSF (#ST506, Beyotime biotechnology, China) and phosphatase inhibitor tablets (#4906837001, Roche, USA). Same quantities of proteins (30 ug) were separated on 10-12.5% SDS page gels and transfected to polyvinylidene difluoride (PVDF) membrane with transfer buffer. After blocking in TBST supplemented with 5% skim milk powder at room temperature for 2 h, membranes were incubated with primary antibodies at 4^o^C overnight, followed by the incubation of Goat anti-rabbit or anti-mouse horseradish peroxidase-conjugated antibodies (1:10,000; respectively Lot 1387291024 and Lot 1357561012, Cell Signaling Technology) for ,at least, 1 h at room temperature. Membranes were washed three to five times during 10 min per washing prior to each step. The primary antibodies used included: GAPDH (1:5,000,#60004-1-Ig,Proteintech,China), TMPRSS4 (1:1,000 dilution,#A4865, ABclonal,USA ), Bax (1:1,000 dilution, #50599-2-Ig, Proteintech,China ), Bcl2 (1:1,000 dilution,#A16776, ABclonal,USA), UBE2D3 (1:1,000 dilution, #11677-1-AP, Proteintech,China), POT1 (1:1,000 dilution, #10581-1-AP, Proteintech,China), TPP1 (1:1,000 dilution,#A5627, ABclonal,USA). The separated proteins bands were visualized using Enhanced chemiluminescence system ECL (Adansta, USA). The images were captured and analyzed (quantified) by respectively using ChemiDoc XRS + System ( Bio-Rad, Hercules, CA,USA) and Image J program. 

*Cell proliferation analysis*

The resultant stably transfected MDA-MB-468 and MCF-7 cells were seeded into the 96 wells plates at 3.103 cells per well in 100 µL DMEM culture medium. At real time post culture for each analysis (0 h, 6 h, 12 h, 24 h, 48 h, and 72 h), CCK-8 (10 μL) was added to each plate and re-incubated at 37^o^C for 90 min. The absorbance of each well was determined at 450 nm (A450 nm) with a microplate reader. Each test was repeated in four wells and performed daily for four days.

*Cell cycle assay*

The resultant stably transfected MDA-MB-468 and MCF-7 cells were seeded into the six wells plates at the 10^5^ /mL cells in 2 mL DMEM medium. At 3 days post-culture, cells were harvested, fixed in 70% ethanol for 30 min and stained with 500 μL of propidium iodure (C1052, Beyotime, China) for 30 min at 37^o^C. Samples were analyzed by flow cytometry (FACS, Aria III, BD, USA). All experiments were performed three times.

*Cell apoptosis assay*

The resultant stably transfected MDA-MB-468 and MCF-7 cells were seeded into the six wells plates at the 10^5^ /mL cells in 2 mL DMEM medium. At 3 days post-culture, cells were collected and apoptosis tests were performing using FITC - Conjugated annexin V / Propidium iodide method (Annexin V- FITC /PI apoptosis kit, Best Bio/China) according to the manufacturer’s instructions. All tests were carried out in triplicate.

*Statistical Analysis*

GraphPad Prism 5.0 was used for Statistical analysis. One way ANOVA was used for comparison of multiple groups with Turkey’s method and p-Value <0.05. Data are presented as the mean ± SD. Value of P<0.05 was considered to indicate a statistically significant difference.

## Results

*Effect of stable transfection in MDA-MB-468 and MCF-7 on normal TMPRSS4 expression*

**Figure 1:**
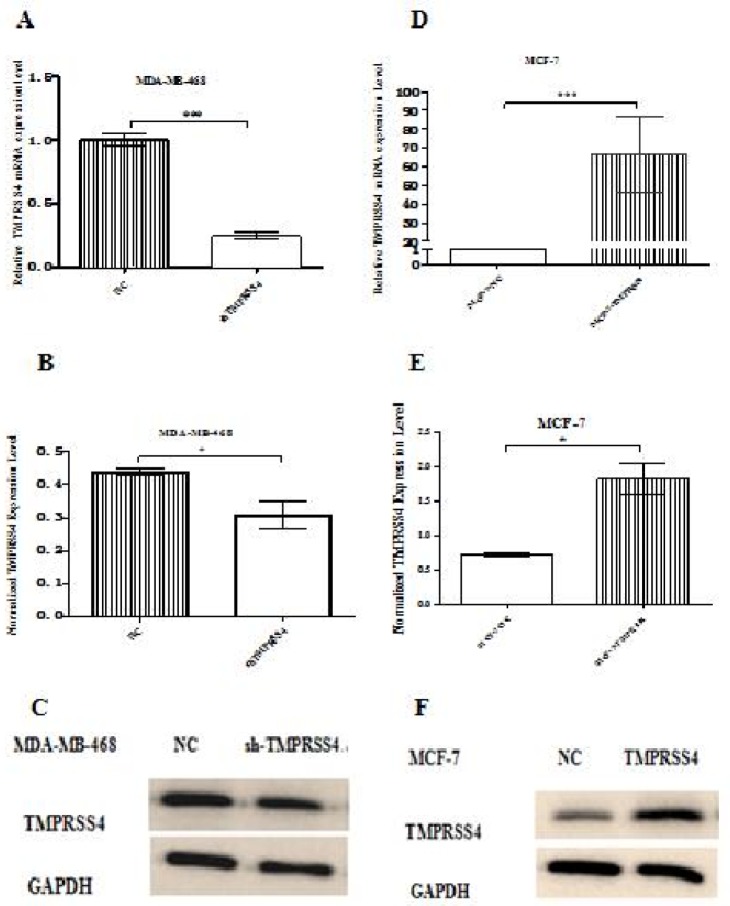
Expression of TMPRSS4 mRNA and Proteins in MDA-MB-468 and MCF-7 Cells Respectively Transfected with Down-Regulating and Up-Regulating TMPRSS4 Vectors. (A, D) qPCR analysis for TMPRSS4 mRNA expression in MDA-MB-468 and MCF-7 cells; (B, C, E, and F) Western blot analysis for TMPRSS4 protein expression in MDA-MB-468 and MCF-7 cells. TMPRSS4 expression was significantly inhibited at mRNA and protein level in MDA-MB-468-shTMPRSS4 versus MDA-MB-468-NC cells [(A) mRNA; ***P<0.001 and (B,C) Protein; *P<0.05 respectively] while TMPRSS4 was significantly overexpressed at mRNA and protein level in MCF-7-TMPRSS4 versus MCF-7-NC [mRNA; ***P<0.001 (D) and Protein; *P<0.05 (E,F), respectively]. Values were indicated as the mean ±SD of three independent experiments

In order to analyze the effect of TMPRSS4 down-regulating and up-regulating on normal TMPRSS4 expression in breast cancer, the MDA-MB-468 and MCF-7 breast cancer cell lines were respectively selected to perform the experiments based on the report of Li et al., (2017) where it has been shown that TMPRSS4 is highly expressed in MDA-MB-468 cells and lowly expressed in MFC-7 cell lines. For confirmation of efficient transfection, qPCR and western blot were respectively used and showed that stable transfection in MDA-MB-468 results to the reduction of TMPRSS4 mRNA (Figure 1A) and TMPRSS4 protein (Figure 1B,C) expression in MDA-MB-468-sh-TMPRSS4 groups compared to control transfected MDA-MB-468-sh-NC groups with ***P<0.001 and *P<0.05, respectively . In the same way, stable transfection in MCF-7 results to the increase of TMPRSS4 mRNA (Figure 1D) and TMPRSS4 protein (Figure 1E,F) expression in MCF-7-TMPRSS4 groups compared to control transfected MCF-7-NC groups with ***P<0.001 and *P<0.05, respectively.

*Effect of TMPRSS4 modulation on breast cancer cells proliferation.*

**Figure 2:**
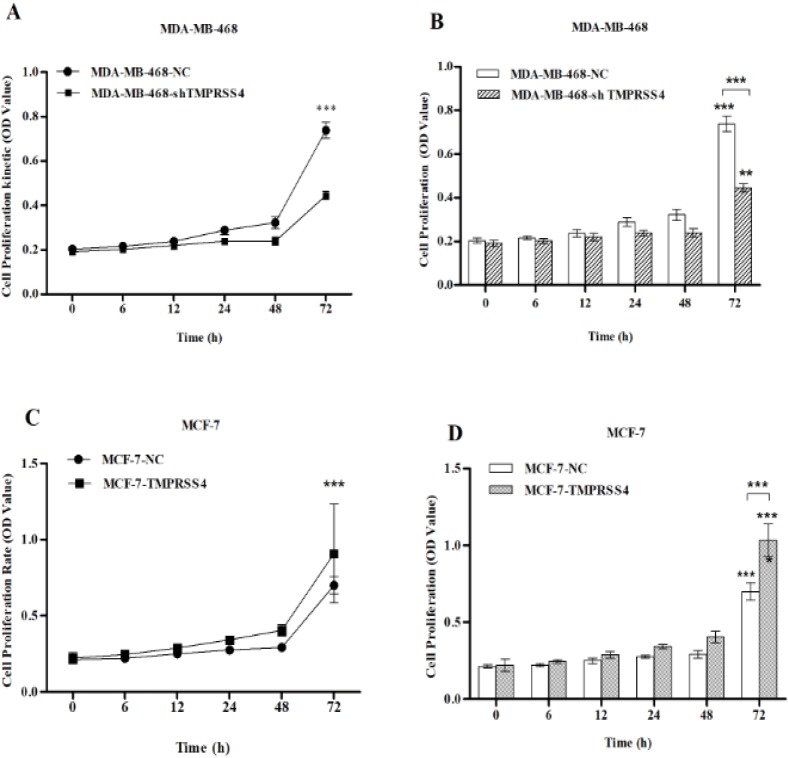
CCK-8 Assay was Used to Investigate the Growth Ability of Stably Transfected MDA-MB-468 and MCF-7 Cells with Down-Regulating and Up-Regulating TMPRSS4 Vectors, Respectively. (A, C) Cells proliferation kinetic during 72 h post-culture, (B, D) Cells proliferation rate at different times of analysis. Down-regulation of TMPRSS4 expression inhibited cells proliferation kinetic and significantly reduced cells proliferation at 72 h post-culture [(A,B); ***P<0.001 at 72 h post-culture].Additionally, TMPRSS4 overexpression increased cell proliferation Kinetic and significantly increased cell proliferation at 72 h post-culture [ (C,D); ***P<0.001 at 72 h post-culture]. Values were indicated as mean ±SD of four independent experiments

Cell proliferation was analyzed by utilizing the CCK-8 assay. The results showed that inhibition of TMPRSS4 expression in MDA-MB-468 cell lines reduced the cell proliferation kinetic (Figure 2A) and significantly decreased cell proliferation at 72 h post-culture in MDA-MB-468-sh-TMPRSS4 groups compare to control transfected MDA-MB-468-sh-NC groups [(45.1±1.94% versus 72.54±1.04%); (Figure 2B, ***P< 0.001)]. Moreover, TMPRSS4 overexpression in MCF-7 cells increases cells proliferation kinetic during 72 h post-culture (Figure 2C) and significantly increases cell proliferation at 72 h post-culture in MCF-7-TMPRSS4 groups compared with MCF-7-NC control transfected groups [(98.84±3,1% versus 69.72±4.7%); (Figure 2D, ***P<0.001).

*Effect of TMPRSS4 modulation on breast cancer cell cycle*

**Figure 3:**
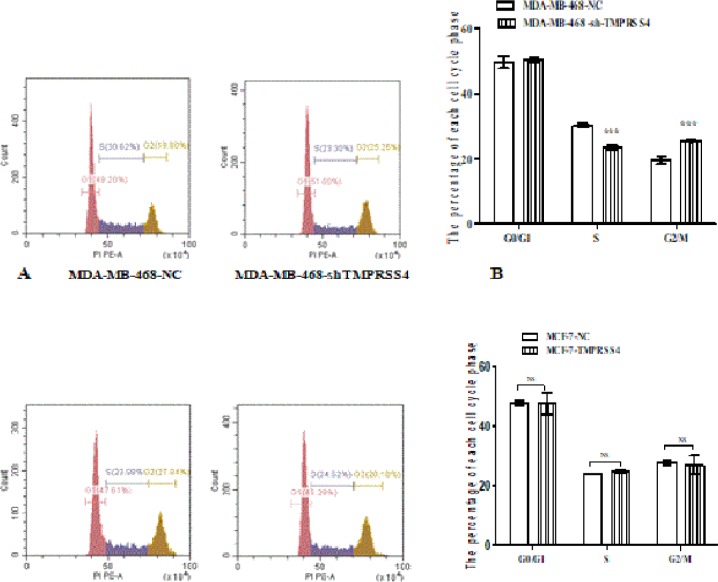
Cell Cycle was Determined by Using Flow Cytometry Analysis in Stably Transfected MDA-MB-468 and MCF-7 Cell Lines. TMPRSS4 expression inhibition significantly promoted the cells arrest in G2 phase and reduced the rate of S phase arrested cells in MDA-MB-468-shTMPRSS4 group compared to the control transfected MDA-MB-468-NC (A, B; *P< 0.001). However, TMPRSS4 over-expression showed no significant changes in cell cycle progression (C, D) when MCF-7-TMPRSS4 it was compared to MCF-7-NC

Cells cycle was examined using flow cytometry (FACS) assay. The results revealed that silencing TMPRSS4 in MDA-MB-468 cells significantly induced cell cycle arrest in G2/M phase [25.63±0.32% versus 19.60±1.21%] and decrease of cell in S phase [ 30.27±0.66% versus 23.52±0.69%], at 72 h post-culture, in MDA-MB-468-sh-TMPRSS4 groups compare to control MDA-MB-468-sh-NC group (Figure 3A,B ; ***P<0.001). However, TMPRSS4 overexpression in MCF-7 cells showed no significant changes in the cells cycle progression of MCF-7-TMPRSS4 compared to MCF-7-NC (Figure 3C,D) at 72 h post-culture.

*Effect of TMPRSS4 modulation on breast cancer cells apoptosis*

**Figure 4:**
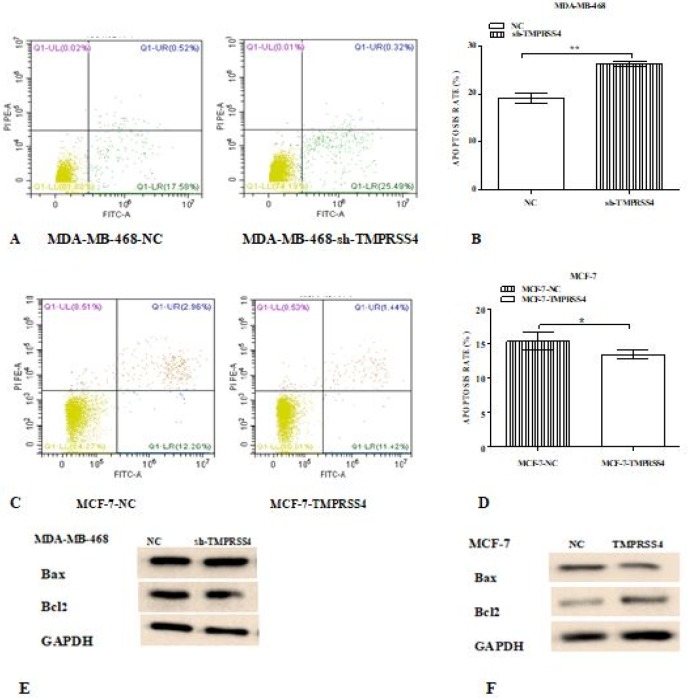
The Annexin-V Staining Assay was Performed to Evaluate Cell Apoptosis in Stably Transfected MDA-MB-468 and MCF-7 Cells According to Manufacturer’s Instruction. After treatment with annexin V, cells were analyzed by using flow cytometry (A - D). Western blot of certain proteins related to apoptosis (E, F) signaling pathway was also performed to assess to apoptosis modulation ability of TMPRSS4 modulation. TMPRSS4 silencing significantly induced apoptosis in stably transfected MDA-MB-468 cells (A and B, **P<0.01) associated with an increase of expression of pro-apoptotic proteins Bax and decrease of expression of anti-apoptotic protein Bcl2 (E). In the same logic, TMPRSS4 overexpression in MCF-7 cells significantly reduced cell apoptosis rate (C and D, *P< 0.05) through a decrease of expression of pro-apoptotic proteins Bax and increase of expression of anti-apoptotic protein Bcl2 (F). All experiments were repeated three times

Cell apoptosis was investigated by performing flow cytometry assay and analyzing the expression of certain proteins (Bcl2 and Bax) related to cell apoptosis signaling pathway. The results showed that TMPRSS4 expression down-regulation increased the rate of apoptotic index in MDA-MB-468-sh-TMPRSS4 groups compared to control MDA-MB-468-sh-NC groups at 72 h post-culture [(26.29±0.62% versus 19.13±1.18%); (Figure 4.AB; **P<0.01)] associated with the increase of pro-apoptotic protein (Bax) and decrease of expression of anti-apoptotic (Bcl2) (Figure 4.E). Otherwise, TMPRSS4 expression up-regulation decreased the rate of apoptotic index in MCF-7-TMPRSS4 groups compared to control transfected MCF-7-NC groups at 72 h post-culture [(13.40±0.71% versus 15.29±1.29%); (Figure 4.C,D; *P<0.05)] associated with the decrease of pro-apoptotic protein ( Bax) and increase of expression of anti-apoptotic (Bcl2) (Figure 4.F).

*Effect of TMPRSS4 modulation on telomere integrity in breast cancer cell lines*

**Figure 5:**
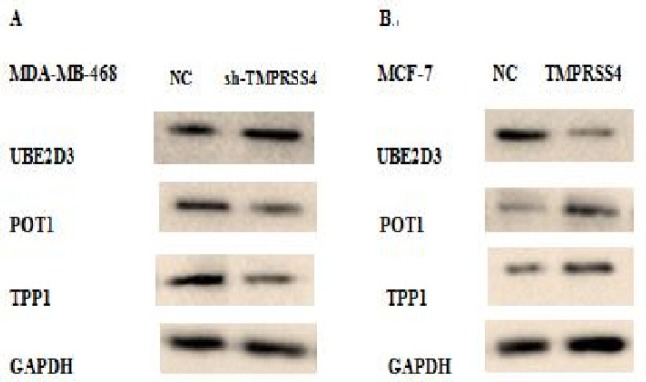
Western Blot Analysis was Performed to Study the Effect of TMPRSS4 Expression Modulation on Telomere Integrity in Stably Transfected MDA-MB-468 and MCF-7 Cell Lines by Analyzing the Expression of Certain Proteins Related to Telomere Maintenance (UBE2D3, POT1, and TPP1). TMPRSS4 expression silencing in MDA-MB-468 cells induces the impairment (reduces) of telomere maintenance via decrease of the expression of certain telosome proteins (POT1, TPP1) and increase of the expression of UBE2D3 protein (A) while the TMPRSS4 overexpression in MCF-7 cells induces the impairment (reinforce) of telomere maintenance via increase of the expression of certain telosome proteins (POT1, TPP1) and decrease of the expression of UBE2D3 protein (B)

Western blot was performed to analyze telomere integrity through certain proteins (POT1, TPP1, and UBE2D3) related to telomere functions. Our findings showed the impairment of expression related to telomere maintenance proteins. TMPRSS4 down-regulation in MDA-MB-468 cells inhibited, in MDA-MB-468-sh-TMPRSS4 groups versus control MDA-MB-468-sh-NC groups, the expression of POT1 and TPP1 and increase expression of UBE2D3 as the proteins positively and negatively related to telomere integrity, respectively (Figure 5.A). Additionally, TMPRSS4 overexpression in MCF-7 cells increases the expression of POT1 and TPP1 and reduced the expression of UBE2D3 in MCF-7-TMPRSS4 groups than in control transfected MCF-7-NC groups (Figure 5.B). Our findings suggest that TMPRSS4 silencing reduces telomere integrity while its up-regulation reinforce telomere maintenance 

## Discussion

The recent studies showed that TMPRSS4, which is kind of TTPs family and frequently overexpressed in the cancer cells (Kim et al.,2014; Chikaishi et al., 2016; Wu et al., 2014; Liang et al., 2013; Cheng et al., 2013), function as a cancer treatment target biomarker through the use of Cancer gene therapy (CGT) strategies. It has been reported that TMPRSS4 silencing reduced cancer proliferation and its overexpression induction increases cell proliferation in many kinds of cancer cell lines (Fan et al., 2018; Huang et al., 2014; Lee et al., 2016; Jung et al., 2008). In the present study, we used MDA-MB-468 triple negative breast cancer which overexpresses TMPRSS4 and MCF-7 triple positive breast cancer cell as a low expressed TMPRSS4 cancer cell (Li et al., 2017) and found that induction of TMPRSS4 expression down-regulation by using down-regulating TMPRSS4 vector inhibited cell proliferation compared to transfected control and the induction of TMPRSS4 up-regulation promotes cell proliferation which confirms the several above mentioned former studies results (Fan et al., 2018; Huang et al., 2014; Lee et al., 2016; Jung et al., 2008). Moreover, the cell cycle is the well-established tumor suppressive mechanism which could be activated to reduce tumor progression and response of cancer to treatment (Daiz Moralli et al., 2013). Regarding the effect of TMPRSS4 on cancer cell cycle, our results showed that TMPRSS4 silencing in MDA-MB-468 breast cancer promotes the cell cycle arrest in G2/M phase associated to the reduction of the cell cycle arrest in S phase while induction of the TMPRSS4 overexpression in MCF-7 breast cancer cell has no significant effect on cell cycle process. This difference results could be due to the expression of estrogen receptor in MFC-7 cell line. In a breast cancer cell, Estrogen Receptor status is inversely associated with a p53 expression which is implicated in cell cycle process perturbation. It has been reported that the sub-unit α of Estrogen receptor (estrogen receptor α) binds to and inactivates p53 (Liu et al., 2006). P53 induces the expression of p21WAF1/CIT1/Sdi1, an inhibitor of the cyclin-dependent kinases (CDKs)2,3,4 and 6 (Shaw et al., 1996).This suggested that the expression of ER in the MCF-7 cell may lead to the lack of the p53 and unchanged of cell cycle process by TMPRSS4. Our findings are in correlation with the report of Jung (Jung et al., 2008) where it has been reported that TMPRSS4 inhibition reduces cell proliferation by down-regulating cyclin D1 and cyclin E1 expression as the genes related to the cell cycle signaling pathways (Jung et al., 2008; Oridate et al., 2005; Kanska et al., 2016), and with the report of Lee et al., (2016) which reported that TMPRSS4 overexpression promotes cell proliferation and induces a 1.89 fold increase of the cell cycle gene cyclin D1 promoter (-962/+134) activity. However, our results contradicted the previous studies which found that the TMPRSS4 silencing reduces cell proliferation with no changes in cell cycle phase (Huang et al., 2014) and the part of the finding of Lee et al., (2016), where it has been reported that, in prostate cancer cell lines, TMPRSS4 overexpression display smaller G0/G1 phase population and larger S phase population compared to control transfected Lee et al., (2016), suggesting that the effect of TMPRSS4 on cell proliferation in relationship with cell cycle is cancer cell type dependent and remains to be more investigated in the future. Otherwise, cell apoptosis is one of the key steps of cancer development and progression and also considered as an anticancer mechanism (Pistritto et al., 2016; Hanahan et al., 2000; Wong et al., 2011; Kadam et al., 2016). The pro-apoptotic (Bax) and anti-apoptotic (Bcl2) proteins are key proteins in the signaling pathway of cell apoptosis and are also used as a therapeutic target for cancer treatment via apoptotic pathway affection (Akl et al., 2016; Adams et al., 2018; Pawlowski et al., 2000; Xin et al., 2014). The present study investigated the link between TMPRSS4 and cell apoptosis in TMPRSS4 modulation mediated cell proliferation modulation and showed that TMPRSS4 silencing reduces cells proliferation by promoting cell apoptosis associated with inhibition of anti-apoptotic protein (Bcl2) and increase of pro-apoptotic (Bax) protein expression whereas TMPRSS4 overexpression induces cells proliferation by reducing cell apoptotic index through increase of anti-apoptotic protein (Bcl2) and decrease of pro-apoptotic protein (Bax) expression. These results confirm the finding of Fan (Fan et al., 2018) where it has been reported that TMPRSS4 modulation promotes cell proliferation modulation by modulating cell apoptosis through Bcl2 expression modulation (Fan et al., 2018) and the findings of Huang et al., (2014) showing that TMPRSS4 silencing reduced cell proliferation via increase of cell apoptosis. Moreover, the telomere is implicated in cancer development and treatment (Bernal et al., 2018; Ivancich et al., 2017). The telosome and Ubiquitin Conjugated Enzyme (UBE2D3) are two kinds of factors implicated in the integrity of telomere (De Lange et al., 2005; Wang et al., 2013). By investigating the relationship between TMPRSS4 and Telomere, we found that TMPRSS4 expression down-regulation reduce the telomere integrity via reduction of expression of POT1, TPP1 as the kind of telosome proteins, and up-regulation of UBE2D3 while TMPRSS4 overexpression reinforces its integrity. As it has been reported in our previous studies that UBE2D3 is negatively correlated with cell proliferation and telomere maintenance (Wang et al., 2013;Yang et al., 2016) and cell proliferation has been inhibited by affecting telomere integrity via silencing of POT1 (Lei et al., 2015;) and TPP1 (Yang et al., 2013), the present study suggest that TMPRSS4 modulation modulates cell proliferation by modulating telomere maintenance in breast cancer cells.

In conclusion, our findings indicate that TMPRSS4 expression modulation modulates breast cancer cell proliferation by modulating cell apoptosis and telomere maintenance while cell cycle is only implicated in TMPRSS4 silencing mediated inhibition of breast cancer cells proliferation. This confirms the implication of TMPRSS4 in cancer progression and can make TMPRSS4 to be considered as a potential therapeutic target for Cancer Gene Therapy (CGT). 

## Data Availability

The analyzed data sets generated during the present study are available from the corresponding author on reasonable request.

## Abbreviations

Bcl-2, B-cell lymphoma2; Bax, Bcl-2-associated X; shRNA, short hairpin RNA; FACS, fluorescence-activated cell sorting; cDNA, complementary DNA; DNA, DeoxyriboNucleic Acid; POP1, Protection of Telomere 1; TPP1, Tripeptidyl Peptidase1; CCK-8, Cell Counting Kit-8 .
